# 7,7-[Ethane-1,2-diylbis(oxy)]-2-[hydroxy(phenyl)methyl]bicyclo[3.3.1]nonan-3-one

**DOI:** 10.1107/S1600536814009040

**Published:** 2014-05-17

**Authors:** Jian Li, Jia Qi Ma, Wei Min Mo, Zhen Lu Shen

**Affiliations:** a College of Chemical Engineering and Materials Science, Zhejiang University of Technology, Hangzhou 310014, People’s Republic of China

## Abstract

In the title compound, C_18_H_22_O_4_, the cyclo­hexane and cyclo­hexa­none rings adopt normal chair and half-chair conformations, respectively. The dioxolane ring is almost planar, with an r.m.s. deviation of 0.094 (3) Å. In the crystal, mol­ecules are connected by O—H⋯O hydrogen bonds, forming 2_1_ helical chains along the *a-*axis direction. The chains are further connected by C—H⋯O hydrogen bonds.

## Related literature   

For related structures having condensed cyclo­hexa­none rings, see: Li *et al.* (2002 ▶); Lopez-Alvarado *et al.* (2002 ▶). For a related structure with a cyclo­hexa­none ring, see: Shallard-Brown *et al.* (2005 ▶). For the synthesis, see: Tomizawa *et al.* (2009 ▶).
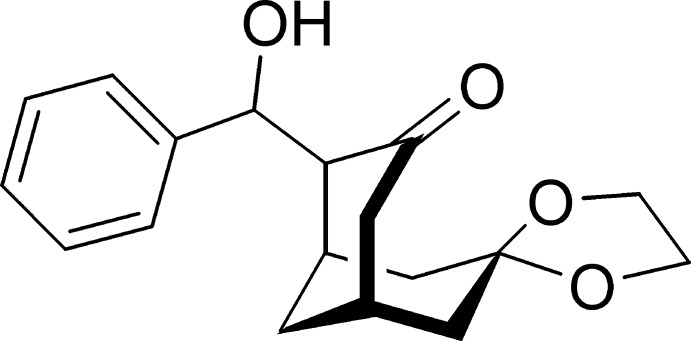


## Experimental   

### 

#### Crystal data  

C_18_H_22_O_4_
*M* *_r_* = 302.36
Orthorhombic, 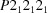

*a* = 9.1465 (3) Å
*b* = 10.0346 (4) Å
*c* = 16.6469 (6) Å
*V* = 1527.88 (10) Å^3^
*Z* = 4
Mo *K*α radiation
μ = 0.09 mm^−1^
*T* = 293 K
0.35 × 0.30 × 0.29 mm

#### Data collection  

Bruker SMART CCD area detector diffractometer
Absorption correction: multi-scan (*SADABS*; Bruker, 2000 ▶) *T*_min_ = 0.951, *T*_max_ = 0.962
12383 measured reflections
1734 independent reflections
1560 reflections with *I* > 2σ(*I*)
*R*_int_ = 0.026

#### Refinement  

*R*[*F* ^2^ > 2σ(*F* ^2^)] = 0.039
*wR*(*F* ^2^) = 0.096
*S* = 1.03
1734 reflections
203 parameters
H atoms treated by a mixture of independent and constrained refinement
Δρ_max_ = 0.20 e Å^−3^
Δρ_min_ = −0.17 e Å^−3^

### 

Data collection: *SMART* (Bruker, 2000 ▶); cell refinement: *SAINT* (Bruker, 2000 ▶); data reduction: *SAINT*; program(s) used to solve structure: *SHELXTL* (Sheldrick, 2008 ▶); program(s) used to refine structure: *SHELXTL*; molecular graphics: *SHELXTL*; software used to prepare material for publication: *SHELXTL*.

## Supplementary Material

Crystal structure: contains datablock(s) global, I. DOI: 10.1107/S1600536814009040/is5345sup1.cif

Structure factors: contains datablock(s) I. DOI: 10.1107/S1600536814009040/is5345Isup2.hkl

CCDC reference: 998726

Additional supporting information:  crystallographic information; 3D view; checkCIF report

## Figures and Tables

**Table 1:** Hydrogen-bond geometry (Å, °)

*D*—H⋯*A*	*D*—H	H⋯*A*	*D*⋯*A*	*D*—H⋯*A*
O4—H4⋯O3^i^	0.86 (4)	2.03 (4)	2.857 (3)	163 (3)
C16—H16⋯O3^ii^	0.93	2.59	3.473 (4)	159

Symmetry codes: (i) 
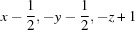
; (ii) 
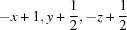
.

**Fig. 1.**
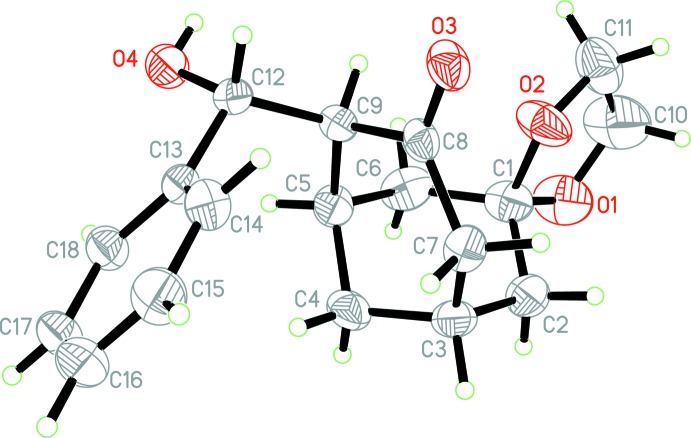
The molecular structure of the title compound with atom labels, showing 40% probability displacement ellipsoids.

**Fig. 2.**
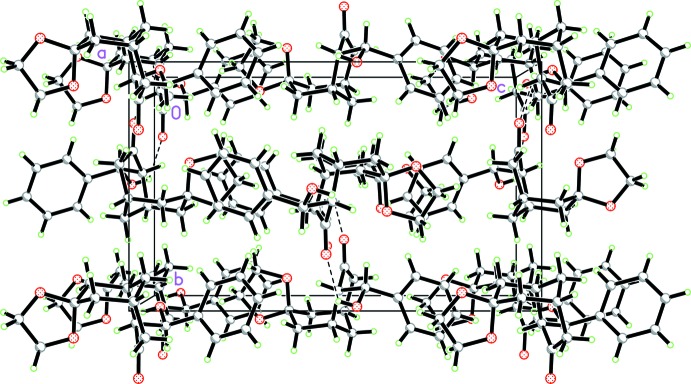
The packing diagram of the title compound viewed down along the a axis. O—H···O hydrogen bonds are shown by thin dashed lines.

## supplementary crystallographic information

### 1. Comment

2-(Hydroxyphenylmethyl)-7,7-ethylenedioxybicyclo[3.3.1]nonan-3-one is a key intermediate of the known asymmetrical catalysis of adamantane derivatives that is used in kinetic resolution of secondary alcohols (Tomizawa *et al.*, 2009). Recently. it was synthesized in our lab and the structure was determined.

In the compound (Fig. 1), the C1/C4 cyclohexyl ring adopts a normal chair conformation. The O1/O2 dioxolane ring is almost planar, with a maximum deviation 0.086 (4) Å at the O2 atom. Atoms C3, C7, C8, C9, C5 and O3 are arranged roughly on a plane, with the maximum deviation 0.171 (4) Å at C7 atom. Therefore, the C4/C8 cyclohexanone ring adopts a half-chair conformation, which is different from most known condensed cyclohexanone ring that is part of the complex structure (Lopez-Alvarado *et al.*, 2002; Li *et al.*, 2002), and from free cyclohexanone in solid state (Shallard-Brown *et al.*, 2005).

In the crystal, molecules are linked by O4—H4···O3^i^ hydrogen bonds (Fig. 2 and Table 1) to form chiral chains. In addition, C—H···O hydrogen bonds help the stability of the crystal. No π–π packing and C—H···π contacts are observed.

### 2. Experimental

2-(Hydroxyphenylmethyl)-7,7-ethylenedioxybicyclo[3.3.1]nonan-3-one was synthesized through a known procedure (Tomizawa *et al.*, 2009) and obtained as a white solid in a yield of 61% and a purity of 96%. Single crystals were obtained by recrystalization from anhydrous ethanol. MS (ESI) 325.1 (*M*+Na+) m/*z*.

### 3. Refinement

The hydroxyl H atom was located in a difference Fourier map and was refined freely. Other H atoms were placed in calculated positions and allowed to ride on their parent atoms at distances 0.93 Å for phenyl, 0.97 Å for methylene and 0.98 Å for methine with *U*_iso_(H) = 1.2*U*_eq_(C). In the absence of significant anomalous scattering effects, Friedel pairs have been merged.

### Crystal data

**Table:**

C_18_H_22_O_4_	*D*_x_ = 1.314 Mg m^−^^3^
*M**_r_* = 302.36	Mo *K*α radiation, λ = 0.71073 Å
Orthorhombic, *P*2_1_2_1_2_1_	Cell parameters from 1165 reflections
*a* = 9.1465 (3) Å	θ = 2.4–24.4°
*b* = 10.0346 (4) Å	µ = 0.09 mm^−^^1^
*c* = 16.6469 (6) Å	*T* = 293 K
*V* = 1527.88 (10) Å^3^	Prism, colorless
*Z* = 4	0.35 × 0.30 × 0.29 mm
*F*(000) = 648	

### Data collection

**Table:**

Bruker SMART CCD area detector diffractometer	1734 independent reflections
Radiation source: fine-focus sealed tube	1560 reflections with *I* > 2σ(*I*)
Graphite monochromator	*R*_int_ = 0.026
φ and ω scans	θ_max_ = 26.0°, θ_min_ = 3.3°
Absorption correction: multi-scan (*SADABS*; Bruker, 2000)	*h* = −10→11
*T*_min_ = 0.951, *T*_max_ = 0.962	*k* = −12→12
12383 measured reflections	*l* = −20→20

### Refinement

**Table:**

Refinement on *F*^2^	Primary atom site location: structure-invariant direct methods
Least-squares matrix: full	Secondary atom site location: difference Fourier map
*R*[*F*^2^ > 2σ(*F*^2^)] = 0.039	Hydrogen site location: inferred from neighbouring sites
*wR*(*F*^2^) = 0.096	H atoms treated by a mixture of independent and constrained refinement
*S* = 1.03	*w* = 1/[σ^2^(*F*_o_^2^) + (0.0446*P*)^2^ + 0.4409*P*] where *P* = (*F*_o_^2^ + 2*F*_c_^2^)/3
1734 reflections	(Δ/σ)_max_ < 0.001
203 parameters	Δρ_max_ = 0.20 e Å^−^^3^
0 restraints	Δρ_min_ = −0.17 e Å^−^^3^

### Special details

**Table:**

Experimental. IR (KBr) 3488, 2929, 2901, 2875, 1685, 1455, 1332, 1268, 1215, 1145, 1094, 1072, 1044, 1024, 948, 715 cm^-1. 1^NMR (CDCl3) 7.28–7.39 (m, 5H), 4.68–4.70(m, 1H), 3.92–3.95(m, 2H), 3.89 (d, J = 1.5 Hz, 1H), 3.80–3.87 (m, 2H), 2.59–2.63 (m, 2H), 2.51–2.55 (m, 2H), 1.96 (s, 1H), 1.80–1.83 (m, 3H), 1.64–1.67 (m, 1H), 1.52–1.57 (m, 2H) p.p.m.. ^13^CNMR (CDCl3) 213.2, 141.3, 128.6, 128.2, 127.1, 107.3, 74.6, 64.4, 63.4, 60.1, 45.1, 41.0, 40.1, 30.5, 29.2, 28.4 p.p.m..
Geometry. All e.s.d.'s (except the e.s.d. in the dihedral angle between two l.s. planes) are estimated using the full covariance matrix. The cell e.s.d.'s are taken into account individually in the estimation of e.s.d.'s in distances, angles and torsion angles; correlations between e.s.d.'s in cell parameters are only used when they are defined by crystal symmetry. An approximate (isotropic) treatment of cell e.s.d.'s is used for estimating e.s.d.'s involving l.s. planes.
Refinement. Refinement of *F*^2^ against ALL reflections. The weighted *R*-factor *wR* and goodness of fit *S* are based on *F*^2^, conventional *R*-factors *R* are based on *F*, with *F* set to zero for negative *F*^2^. The threshold expression of *F*^2^ > σ(*F*^2^) is used only for calculating *R*-factors(gt) *etc*. and is not relevant to the choice of reflections for refinement. *R*-factors based on *F*^2^ are statistically about twice as large as those based on *F*, and *R*- factors based on ALL data will be even larger.

### Fractional atomic coordinates and isotropic or equivalent isotropic displacement parameters (Å^2^)

**Table:**

	*x*	*y*	*z*	*U*_iso_*/*U*_eq_	
O1	0.6491 (3)	0.0825 (2)	0.69986 (12)	0.0753 (7)	
O2	0.5859 (3)	−0.1011 (2)	0.62676 (11)	0.0652 (6)	
O3	0.4901 (2)	−0.27188 (15)	0.47755 (10)	0.0465 (5)	
O4	0.1336 (2)	−0.00830 (19)	0.44707 (11)	0.0497 (5)	
C1	0.6058 (3)	0.0396 (3)	0.62152 (15)	0.0506 (7)	
C2	0.7289 (3)	0.0663 (3)	0.56347 (16)	0.0496 (7)	
H2A	0.8089	0.0059	0.5752	0.060*	
H2B	0.7640	0.1565	0.5717	0.060*	
C3	0.6849 (3)	0.0497 (3)	0.47526 (15)	0.0454 (6)	
H3	0.7658	0.0822	0.4420	0.054*	
C4	0.5513 (3)	0.1339 (3)	0.45651 (16)	0.0484 (6)	
H4A	0.5262	0.1253	0.4001	0.058*	
H4B	0.5720	0.2269	0.4675	0.058*	
C5	0.4239 (3)	0.0869 (2)	0.50846 (16)	0.0428 (6)	
H5	0.3392	0.1433	0.4965	0.051*	
C6	0.4659 (3)	0.1088 (3)	0.59727 (17)	0.0550 (7)	
H6A	0.4764	0.2037	0.6069	0.066*	
H6B	0.3869	0.0767	0.6310	0.066*	
C7	0.6542 (3)	−0.0947 (3)	0.45113 (15)	0.0431 (6)	
H7A	0.6618	−0.1010	0.3931	0.052*	
H7B	0.7305	−0.1503	0.4738	0.052*	
C8	0.5093 (3)	−0.1518 (2)	0.47572 (13)	0.0351 (5)	
C9	0.3807 (3)	−0.0595 (2)	0.49186 (13)	0.0340 (5)	
H9	0.3354	−0.0919	0.5415	0.041*	
C10	0.6360 (6)	−0.0235 (4)	0.75276 (19)	0.0986 (15)	
H10A	0.5687	−0.0013	0.7958	0.118*	
H10B	0.7303	−0.0448	0.7762	0.118*	
C11	0.5806 (4)	−0.1374 (4)	0.70659 (17)	0.0695 (10)	
H11A	0.6407	−0.2155	0.7160	0.083*	
H11B	0.4809	−0.1578	0.7222	0.083*	
C12	0.2635 (2)	−0.0783 (2)	0.42468 (14)	0.0361 (5)	
H12	0.2395	−0.1734	0.4220	0.043*	
C13	0.3099 (2)	−0.0343 (2)	0.34160 (13)	0.0351 (5)	
C14	0.3927 (3)	−0.1185 (3)	0.29428 (14)	0.0445 (6)	
H14	0.4169	−0.2028	0.3133	0.053*	
C15	0.4402 (3)	−0.0792 (3)	0.21877 (16)	0.0559 (7)	
H15	0.4965	−0.1369	0.1878	0.067*	
C16	0.4044 (3)	0.0444 (3)	0.18965 (17)	0.0598 (8)	
H16	0.4362	0.0709	0.1391	0.072*	
C17	0.3212 (3)	0.1284 (3)	0.23589 (17)	0.0541 (7)	
H17	0.2968	0.2123	0.2163	0.065*	
C18	0.2731 (3)	0.0903 (3)	0.31126 (15)	0.0437 (6)	
H18	0.2161	0.1481	0.3417	0.052*	
H4	0.078 (4)	−0.061 (4)	0.474 (2)	0.085 (12)*	

### Atomic displacement parameters (Å^2^)

**Table:**

	*U*^11^	*U*^22^	*U*^33^	*U*^12^	*U*^13^	*U*^23^
O1	0.1027 (18)	0.0848 (16)	0.0384 (10)	−0.0320 (15)	−0.0114 (11)	−0.0115 (11)
O2	0.1016 (17)	0.0530 (12)	0.0411 (10)	−0.0264 (12)	−0.0194 (11)	0.0094 (9)
O3	0.0556 (11)	0.0299 (9)	0.0539 (10)	0.0049 (8)	−0.0135 (9)	−0.0010 (8)
O4	0.0375 (9)	0.0539 (12)	0.0576 (11)	0.0058 (9)	0.0073 (9)	0.0130 (9)
C1	0.0665 (18)	0.0489 (15)	0.0363 (12)	−0.0176 (14)	−0.0052 (12)	−0.0066 (11)
C2	0.0489 (15)	0.0500 (15)	0.0500 (14)	−0.0117 (13)	−0.0108 (12)	−0.0009 (12)
C3	0.0436 (13)	0.0515 (15)	0.0411 (12)	−0.0118 (12)	0.0011 (11)	0.0048 (12)
C4	0.0596 (15)	0.0357 (13)	0.0499 (14)	−0.0120 (12)	−0.0102 (12)	0.0089 (11)
C5	0.0437 (13)	0.0309 (12)	0.0538 (14)	0.0018 (11)	−0.0048 (11)	−0.0021 (11)
C6	0.0579 (17)	0.0506 (16)	0.0564 (16)	−0.0087 (14)	0.0072 (13)	−0.0189 (13)
C7	0.0390 (13)	0.0511 (15)	0.0391 (12)	0.0030 (12)	−0.0009 (10)	−0.0044 (11)
C8	0.0421 (12)	0.0352 (12)	0.0281 (10)	0.0014 (10)	−0.0074 (10)	0.0013 (9)
C9	0.0390 (12)	0.0311 (11)	0.0320 (10)	−0.0010 (10)	0.0005 (9)	0.0025 (9)
C10	0.128 (4)	0.126 (4)	0.0412 (16)	−0.041 (3)	−0.009 (2)	0.002 (2)
C11	0.082 (2)	0.079 (2)	0.0473 (16)	0.022 (2)	0.0053 (16)	0.0114 (16)
C12	0.0329 (12)	0.0333 (12)	0.0422 (12)	−0.0030 (10)	−0.0008 (10)	0.0050 (10)
C13	0.0329 (12)	0.0359 (12)	0.0365 (11)	−0.0049 (10)	−0.0065 (9)	0.0017 (10)
C14	0.0470 (14)	0.0447 (14)	0.0420 (12)	0.0035 (12)	−0.0029 (11)	−0.0007 (11)
C15	0.0582 (17)	0.0692 (19)	0.0403 (14)	0.0010 (16)	0.0036 (12)	−0.0045 (14)
C16	0.0600 (18)	0.080 (2)	0.0393 (13)	−0.0102 (17)	0.0024 (13)	0.0139 (15)
C17	0.0601 (17)	0.0504 (16)	0.0518 (15)	−0.0063 (14)	−0.0077 (13)	0.0182 (13)
C18	0.0473 (14)	0.0395 (13)	0.0445 (13)	−0.0006 (11)	−0.0043 (11)	0.0046 (11)

### Geometric parameters (Å, º)

**Table:**

O1—C10	1.386 (4)	C7—H7A	0.9700
O1—C1	1.429 (3)	C7—H7B	0.9700
O2—C11	1.379 (3)	C8—C9	1.521 (3)
O2—C1	1.425 (3)	C9—C12	1.561 (3)
O3—C8	1.218 (3)	C9—H9	0.9800
O4—C12	1.430 (3)	C10—C11	1.467 (5)
O4—H4	0.86 (4)	C10—H10A	0.9700
C1—C2	1.508 (4)	C10—H10B	0.9700
C1—C6	1.511 (4)	C11—H11A	0.9700
C2—C3	1.532 (4)	C11—H11B	0.9700
C2—H2A	0.9700	C12—C13	1.513 (3)
C2—H2B	0.9700	C12—H12	0.9800
C3—C4	1.518 (4)	C13—C14	1.381 (3)
C3—C7	1.529 (4)	C13—C18	1.390 (3)
C3—H3	0.9800	C14—C15	1.387 (4)
C4—C5	1.526 (4)	C14—H14	0.9300
C4—H4A	0.9700	C15—C16	1.371 (4)
C4—H4B	0.9700	C15—H15	0.9300
C5—C6	1.543 (4)	C16—C17	1.372 (4)
C5—C9	1.546 (3)	C16—H16	0.9300
C5—H5	0.9800	C17—C18	1.384 (4)
C6—H6A	0.9700	C17—H17	0.9300
C6—H6B	0.9700	C18—H18	0.9300
C7—C8	1.500 (3)		
			
C10—O1—C1	108.9 (2)	O3—C8—C7	120.8 (2)
C11—O2—C1	109.0 (2)	O3—C8—C9	119.1 (2)
C12—O4—H4	109 (2)	C7—C8—C9	119.91 (19)
O2—C1—O1	106.1 (2)	C8—C9—C5	114.35 (19)
O2—C1—C2	108.1 (2)	C8—C9—C12	109.31 (18)
O1—C1—C2	108.9 (2)	C5—C9—C12	114.77 (18)
O2—C1—C6	111.3 (2)	C8—C9—H9	105.9
O1—C1—C6	109.9 (2)	C5—C9—H9	105.9
C2—C1—C6	112.3 (2)	C12—C9—H9	105.9
C1—C2—C3	113.5 (2)	O1—C10—C11	107.1 (3)
C1—C2—H2A	108.9	O1—C10—H10A	110.3
C3—C2—H2A	108.9	C11—C10—H10A	110.3
C1—C2—H2B	108.9	O1—C10—H10B	110.3
C3—C2—H2B	108.9	C11—C10—H10B	110.3
H2A—C2—H2B	107.7	H10A—C10—H10B	108.5
C4—C3—C7	109.0 (2)	O2—C11—C10	106.7 (3)
C4—C3—C2	110.3 (2)	O2—C11—H11A	110.4
C7—C3—C2	113.8 (2)	C10—C11—H11A	110.4
C4—C3—H3	107.8	O2—C11—H11B	110.4
C7—C3—H3	107.8	C10—C11—H11B	110.4
C2—C3—H3	107.8	H11A—C11—H11B	108.6
C3—C4—C5	109.0 (2)	O4—C12—C13	109.15 (19)
C3—C4—H4A	109.9	O4—C12—C9	108.93 (19)
C5—C4—H4A	109.9	C13—C12—C9	115.27 (18)
C3—C4—H4B	109.9	O4—C12—H12	107.7
C5—C4—H4B	109.9	C13—C12—H12	107.7
H4A—C4—H4B	108.3	C9—C12—H12	107.7
C4—C5—C6	108.0 (2)	C14—C13—C18	118.4 (2)
C4—C5—C9	112.8 (2)	C14—C13—C12	119.8 (2)
C6—C5—C9	111.7 (2)	C18—C13—C12	121.8 (2)
C4—C5—H5	108.1	C13—C14—C15	121.0 (3)
C6—C5—H5	108.1	C13—C14—H14	119.5
C9—C5—H5	108.1	C15—C14—H14	119.5
C1—C6—C5	113.7 (2)	C16—C15—C14	120.1 (3)
C1—C6—H6A	108.8	C16—C15—H15	119.9
C5—C6—H6A	108.8	C14—C15—H15	119.9
C1—C6—H6B	108.8	C15—C16—C17	119.4 (3)
C5—C6—H6B	108.8	C15—C16—H16	120.3
H6A—C6—H6B	107.7	C17—C16—H16	120.3
C8—C7—C3	116.9 (2)	C16—C17—C18	121.0 (3)
C8—C7—H7A	108.1	C16—C17—H17	119.5
C3—C7—H7A	108.1	C18—C17—H17	119.5
C8—C7—H7B	108.1	C17—C18—C13	120.1 (3)
C3—C7—H7B	108.1	C17—C18—H18	119.9
H7A—C7—H7B	107.3	C13—C18—H18	119.9
			
C11—O2—C1—O1	14.4 (4)	O3—C8—C9—C12	−64.0 (3)
C11—O2—C1—C2	131.1 (3)	C7—C8—C9—C12	110.9 (2)
C11—O2—C1—C6	−105.2 (3)	C4—C5—C9—C8	37.9 (3)
C10—O1—C1—O2	−8.1 (4)	C6—C5—C9—C8	−83.9 (3)
C10—O1—C1—C2	−124.2 (3)	C4—C5—C9—C12	−89.6 (2)
C10—O1—C1—C6	112.4 (3)	C6—C5—C9—C12	148.6 (2)
O2—C1—C2—C3	76.3 (3)	C1—O1—C10—C11	−0.8 (5)
O1—C1—C2—C3	−168.8 (2)	C1—O2—C11—C10	−14.8 (4)
C6—C1—C2—C3	−46.9 (3)	O1—C10—C11—O2	9.6 (5)
C1—C2—C3—C4	53.7 (3)	C8—C9—C12—O4	170.56 (19)
C1—C2—C3—C7	−69.2 (3)	C5—C9—C12—O4	−59.4 (3)
C7—C3—C4—C5	64.1 (3)	C8—C9—C12—C13	−66.4 (2)
C2—C3—C4—C5	−61.5 (3)	C5—C9—C12—C13	63.6 (3)
C3—C4—C5—C6	62.2 (3)	O4—C12—C13—C14	−154.8 (2)
C3—C4—C5—C9	−61.7 (3)	C9—C12—C13—C14	82.3 (3)
O2—C1—C6—C5	−72.3 (3)	O4—C12—C13—C18	26.0 (3)
O1—C1—C6—C5	170.5 (2)	C9—C12—C13—C18	−96.9 (3)
C2—C1—C6—C5	49.1 (3)	C18—C13—C14—C15	1.1 (4)
C4—C5—C6—C1	−56.8 (3)	C12—C13—C14—C15	−178.2 (2)
C9—C5—C6—C1	67.8 (3)	C13—C14—C15—C16	−0.5 (4)
C4—C3—C7—C8	−45.7 (3)	C14—C15—C16—C17	0.0 (4)
C2—C3—C7—C8	77.9 (3)	C15—C16—C17—C18	0.0 (4)
C3—C7—C8—O3	−161.2 (2)	C16—C17—C18—C13	0.6 (4)
C3—C7—C8—C9	24.0 (3)	C14—C13—C18—C17	−1.1 (4)
O3—C8—C9—C5	165.8 (2)	C12—C13—C18—C17	178.1 (2)
C7—C8—C9—C5	−19.3 (3)		

### Hydrogen-bond geometry (Å, º)

**Table:**

*D*—H···*A*	*D*—H	H···*A*	*D*···*A*	*D*—H···*A*
O4—H4···O3^i^	0.86 (4)	2.03 (4)	2.857 (3)	163 (3)
C16—H16···O3^ii^	0.93	2.59	3.473 (4)	159

Symmetry codes: (i) *x*−1/2, −*y*−1/2, −*z*+1; (ii) −*x*+1, *y*+1/2, −*z*+1/2.
